# Outcomes in Patients With Peripheral Arterial Disease With and Without Prior Minor Amputation: A Comparative Analysis

**DOI:** 10.7759/cureus.111112

**Published:** 2026-06-18

**Authors:** Rakibul Hasan, Nur A Al Amin, Mainul Mahmud, Sourav Bhowmick, Santo Barman, Redoy Ranjan

**Affiliations:** 1 Vascular Surgery, Bangladesh Medical University, Dhaka, BGD; 2 Vascular Surgery, Ibn Sina Specialized Hospital, Dhaka, BGD; 3 Vascular Surgery, SIBL Foundation Hospital, Dhaka, BGD; 4 Medicine, Mugda Medical College and Hospital, Dhaka, BGD; 5 Cardiac Surgery, St George's University Hospitals NHS Foundation Trust, London, GBR; 6 Cardiac Surgery, Bangabandhu Sheikh Mujib Medical University, Dhaka, BGD; 7 Biological Science, Royal Holloway University of London, London, GBR

**Keywords:** major amputation, major limb amputation, minor amputation, peripheral arterial disease. chronic limb-threatening ischaemia, peripheral arterial disease (pad), severe peripheral arterial disease

## Abstract

Background: Peripheral arterial disease (PAD) is a major global health concern, as critical limb-threatening ischemia leads to higher rates of amputation and mortality. The prognostic impact of prior minor amputation (PMA) is unclear. This study compares major amputation and all-cause mortality rates in patients with PAD with and without PMA and examines key demographic and clinical differences between these groups.

Methods: This retrospective cohort study included 430 consecutive patients with PAD at a tertiary vascular center. Patients were stratified according to PMA status into a PMA group (*n* = 72, 16.7%) and a non-PMA group (*n* = 358, 83.3%). Demographic data, comorbidities, and treatments were analyzed. Primary outcomes were major amputation and all-cause mortality. Statistical analysis included unpaired t-tests, chi-square tests, Cox proportional hazards regression, and Kaplan-Meier analysis.

Results: Among 430 patients, 72 (16.7%) had PMA, whereas 358 (83.3%) did not. The PMA group had higher rates of smoking (*n* = 45, 62.5%, vs. *n* = 175, 48.9%; *P* = 0.039), chronic kidney disease (*n* = 29, 40.3%, vs. *n* = 98, 27.4%; *P* = 0.034), and revascularization (*n* = 16, 22.2%, vs. *n* = 14, 3.9%; *P* < 0.001), but lower utilization of antiplatelet therapy (*n* = 34, 47.2%, vs. *n* = 229, 64.0%; *P* = 0.006) and statin therapy (*n* = 38, 52.8%, vs. *n* = 258, 72.1%; *P* = 0.001). Although unadjusted Kaplan-Meier analysis demonstrated lower survival in the PMA group, multivariable Cox regression showed that PMA was associated with a lower adjusted risk of major amputation (hazard ratio (HR), 0.05; 95% confidence interval (CI), 0.02-0.18; *P* = 0.001) and all-cause mortality (HR, 0.44; 95% CI, 0.22-0.87; *P* = 0.017). Statin therapy (HR, 0.21; 95% CI, 0.10-0.43; *P* < 0.001) and anticoagulation (HR, 0.39; 95% CI, 0.21-0.72; *P* = 0.003) were associated with reduced mortality risk, whereas revascularization was associated with increased mortality risk (HR, 2.02; 95% CI, 1.05-3.83; *P* = 0.033).

Conclusions: PMA was associated with lower adjusted risks of major amputation and mortality in this retrospective cohort of patients with PAD. These results represent observational associations and require prospective validation. Aggressive medical management should be prioritized in all patients with PAD.

## Introduction

Peripheral arterial disease (PAD) represents a significant global health burden, affecting over 200 million individuals worldwide and contributing substantially to cardiovascular morbidity and mortality [1,2]. PAD is characterized by atherosclerotic narrowing of the lower extremity arteries, leading to reduced blood flow and tissue perfusion [3]. The disease spectrum ranges from asymptomatic presentations to critical limb-threatening ischemia (CLTI), with the latter carrying devastating consequences including tissue loss, amputation, and death [2,3]. Minor amputations, defined as amputations distal to the ankle joint, are frequently performed as part of the management strategy for infected or necrotic tissue in patients with PAD [4]. However, the presence of prior minor amputation (PMA) may serve as a marker of advanced disease severity and potentially influence subsequent clinical outcomes [5,6].

The economic burden of PAD is substantial, with annual health care expenditures in individuals with PAD far exceeding those in adults without PAD and contributing to billions of dollars in national health care costs [7]. Major amputation, in particular, is associated with significant incremental healthcare expenditures, prolonged hospitalizations, and reduced quality of life [8, 9]. Mortality rates following major amputation remain alarmingly high, with studies reporting 30-day mortality rates ranging from 5% to 22% and one-year mortality rates approaching 50% in certain populations [10,11]. Despite advances in revascularization techniques and medical management, amputation-related morbidity and mortality continue to pose significant clinical challenges [12,13].

Understanding the prognostic implications of PMA in patients with PAD is crucial for risk stratification, treatment planning, and patient counseling [5,14,15]. PMA may reflect more advanced atherosclerotic disease, greater tissue loss, or inadequate revascularization, all of which could influence the risk of subsequent major amputation and mortality [16,17].

This retrospective observational study aims to compare clinical outcomes, including major amputation and all-cause mortality, in patients with PAD with and without PMA, while identifying demographic and clinical factors that distinguish these patient populations. Due to its observational design, this study is intended to generate hypotheses rather than provide confirmatory evidence.

## Materials and methods

Study design and participants

This retrospective single-center cohort study was conducted at a tertiary care vascular center from June 2021 to December 2025. Consecutive eligible patients were included to minimize selection bias, with exclusions limited to predefined criteria. Inclusion criteria encompassed adult patients (age ≥18 years) with documented PAD confirmed by non-invasive vascular testing (ankle-brachial index <0.9, toe-brachial index <0.7, or imaging evidence of significant arterial stenosis), irrespective of symptom status [18]. Exclusion criteria included traumatic amputations, malignancy-related amputations, acute limb ischemia requiring emergency intervention, and incomplete medical records. Demographic data, comorbidities, and treatment modalities were extracted from electronic medical records through systematic chart review. The study population comprised 430 consecutive patients diagnosed with PAD who presented for evaluation and management. Patients were stratified into two groups based on the presence or absence of documented PMA at initial presentation: the PAD with PMA group (*n* = 72, 16.7%) and the PAD without PMA group (*n* = 358, 83.3%). PMA was defined as any documented toe or partial foot amputation performed before the index vascular presentation. The interval between prior amputation and presentation was inconsistently recorded in the retrospective data. The variable interval between PMA and study entry introduces potential survivor bias, as patients in the PMA group were required to survive both the initial amputation and the intervening period before cohort entry. Inconsistent recording of the timing between prior amputation and presentation prevented assessment of exposure duration, which may have influenced the observed associations.

Ethical consideration

The Institutional Review Board of Bangabandhu Sheikh Mujib Medical University, Dhaka, Bangladesh, approved the study protocol (Ref: BSMMU/2021/9095; IRB approval number: 3651). Informed consent was obtained, and data were encrypted to maintain anonymity. The study was conducted in accordance with the Declaration of Helsinki.

Study variables

Baseline demographic variables included age, gender, and cardiovascular risk factors. Comorbidities assessed included diabetes mellitus (DM), smoking history, myocardial infarction, congestive heart failure (CHF), chronic obstructive pulmonary disease (COPD), and chronic kidney disease (CKD). Treatment interventions recorded included open surgical revascularization, endovascular revascularization, antiplatelet therapy, statin therapy, and anticoagulation [19]. The primary outcomes of interest were major amputation (defined as amputation at or above the ankle level) and all-cause mortality during the follow-up period. Patients were observed from their initial presentation through to major amputation, death, final clinical contact, or the end of the study period. Outcome assessments were conducted for up to 24 months.

Statistical analysis

Statistical analysis was performed using appropriate software packages. Continuous variables were expressed as mean ± standard deviation and compared between groups using unpaired t-tests. Categorical variables were presented as frequencies and percentages and compared using the chi-square test or Fisher’s exact test, as appropriate. Cox proportional hazards regression analysis was used to identify independent predictors of major amputation and all-cause mortality. Age was included in the model as a dichotomous variable (>40 years vs. ≤40 years), distinguishing between relatively premature and more typical age-related presentations of PAD. A categorical approach was chosen to maintain model stability due to the limited number of outcome events, although continuous age modeling may preserve additional prognostic information. Variable selection was based on both univariate significance (*P *< 0.05) and clinical relevance. Because of the limited number of outcome events, the final multivariable model was restricted to a parsimonious set of variables most relevant to outcome prediction to minimize the risk of overfitting. Results are reported as hazard ratios (HRs) with 95% confidence intervals (CIs). Kaplan-Meier (KM) survival analysis was utilized to estimate freedom from major amputation and overall survival, with log-rank tests used to compare survival curves between groups. A *P*-value <0.05 was considered statistically significant for all analyses.

## Results

The study cohort comprised 430 patients with PAD, with 72 (16.7%) having PMA and 358 (83.3%) without PMA. Baseline demographic analysis revealed no significant difference in mean age between groups (52.85 ± 14.93 years in the PMA group vs. 50.28 ± 14.94 years in the non-PMA group, *P *= 0.184). However, significant gender differences were observed, with 41 males (56.9%) in the PMA group compared with 259 males (72.3%) in the non-PMA group (*P* = 0.001, χ² = 10.54). The prevalence of DM was similar between groups (*n* = 42, 58.3% vs. *n* = 211, 58.9%; *P* = 1.000), whereas smoking was significantly more prevalent in the PMA group (*n* = 45, 62.5% vs. *n* = 175, 48.9%; *P* = 0.039, χ² = 4.27). CKD was significantly more common in patients with PMA (*n* = 29, 40.3% vs. *n* = 98, 27.4%; *P* = 0.034, χ² = 4.49). Revascularization patterns differed markedly between groups. Open surgical revascularization was performed in 8 patients (11.1%) in the PMA group compared with 4 patients (1.1%) in the non-PMA group (*P* = 0.01, χ² = 6.63). Endovascular revascularization showed an even more pronounced difference, with 16 patients (22.2%) in the PMA group undergoing endovascular procedures compared with 14 patients (3.9%) in the non-PMA group (*P* < 0.001, χ² = 15.92). Medical management also varied significantly, with antiplatelet therapy utilized less frequently in the PMA group (*n* = 34, 47.2% vs. *n* = 229, 64.0%; *P* = 0.006, χ² = 7.56) and statin therapy similarly underutilized (*n* = 38, 52.8% vs. *n* = 258, 72.1%; *P* = 0.001, χ² = 10.91) (Table 1).

**Table 1:** Demographic characteristics of the patients with PAD with and without prior minor amputation (n = 430). *P* < 0.05 was considered statistically significant. *P*-values were derived from the unpaired t-test, chi-square test, or Fisher's exact test, as appropriate. PAD with PMA, peripheral arterial disease with prior minor amputation; DM, diabetes mellitus; COPD, chronic obstructive pulmonary disease; CKD, chronic kidney disease

Parameters	PAD with PMA (*n *= 72), *n* (%)	PAD without PMA (*n *= 358), *n* (%)	Total, *n* (%)	*P*-value	Test statistics
Age (years), mean ± SD	52.85 ± 14.93	50.28 ± 14.94	50.71 ± 14.93	0.184	*t* = -1.33
Gender (Male)	41 (56.9)	259 (72.3)	300 (69.8)	0.001	χ² = 10.54
DM	42 (58.3)	211 (58.9)	253 (58.8)	1.000	χ² = 0.00
Smoking	45 (62.5)	175 (48.9)	220 (51.2)	0.039	χ² = 4.27
Myocardial infarction	9 (12.5)	46 (12.8)	55 (12.8)	1.000	χ² = 0.01
Congestive heart failure	17 (23.6)	90 (25.1)	107 (24.9)	0.882	χ² = 0.02
COPD	13 (18.1)	88 (24.6)	101 (23.5)	0.287	χ² = 1.13
CKD	29 (40.3)	98 (27.4)	127 (29.5)	0.034	χ² = 4.49
Open revascularization	8 (11.1)	4 (1.1)	12 (2.8)	0.01	χ² = 6.63
Endovascular revascularization	16 (22.2)	14 (3.9)	30 (7.0)	<0.001	χ² = 15.92
Antiplatelet therapy	34 (47.2)	229 (64.0)	263 (61.2)	0.006*	χ² = 7.56
Statin therapy	38 (52.8)	258 (72.1)	296 (68.8)	0.001*	χ² = 10.91
Anticoagulation	27 (37.5)	178 (49.7)	209 (48.6)	0.183	χ² = 1.77

Over the 24-month follow-up period, major amputation was performed in 10 patients: 7 (9.7%) in the PMA group and 3 (0.8%) in the non-PMA group. All-cause mortality was observed in 23 patients, comprising 7 (9.7%) in the PMA group and 16 (4.5%) in the non-PMA group. The composite endpoint of major amputation or death occurred in 26 patients, with 9 (12.5%) in the PMA group and 17 (4.7%) in the non-PMA group.

Cox proportional hazards regression analysis identified several independent predictors of outcomes (Table 2). For major amputation, PMA emerged as a highly significant protective factor (HR 0.05, 95% CI 0.02-0.18, *P* = 0.001), indicating that patients with PMA had a 95% lower risk of subsequent major amputation. Age greater than 40 years showed a trend toward protection (HR 0.23, 95% CI 0.05-1.02, *P* = 0.05). For all-cause mortality, PMA remained protective (HR 0.44, 95% CI 0.22-0.87, *P* = 0.017), while age >40 years was strongly protective (HR 0.29, 95% CI 0.13-0.62, *P* = 0.002). Revascularization was associated with increased mortality risk (HR 2.02, 95% CI 1.05-3.83, *P* = 0.033), while statin therapy (HR 0.21, 95% CI 0.10-0.43, *P *< 0.001) and anticoagulation (HR 0.39, 95% CI 0.21-0.72, *P* = 0.003) were associated with significantly reduced mortality risk.

**Table 2:** Age- and sex-adjusted multivariable Cox proportional hazards regression for long-term major amputation and all-cause mortality. Independent variables with a *P*-value < 0.05 in the univariate analysis presented in Table 1 (age > 40 years, male sex, smoking, chronic kidney disease, prior minor amputation, revascularization, statin therapy, and anticoagulation) were included in the Cox regression analysis. P < 0.05 was considered statistically significant. A dash (-) indicates that no value was available. HR, hazard ratio

Parameters	Major amputation, HR (95% CI)	Major amputation, *P*-value	All-cause mortality, HR (95% CI)	All-cause mortality, *P*-value
Age (>40 years)	0.23 (0.05-1.02)	0.05	0.29 (0.13-0.62)	0.002
Gender (Male)	0.44 (0.10-1.97)	0.290	0.94 (0.49-1.81)	0.85
Prior amputation	0.05 (0.02-0.18)	0.001	0.44 (0.22-0.87)	0.017
Revascularization	-	-	2.02 (1.05-3.83)	0.033
Statin therapy	-	-	0.21 (0.10-0.43)	<0.001
Anticoagulation	-	-	0.39 (0.21-0.72)	0.003

**Figure 1:**
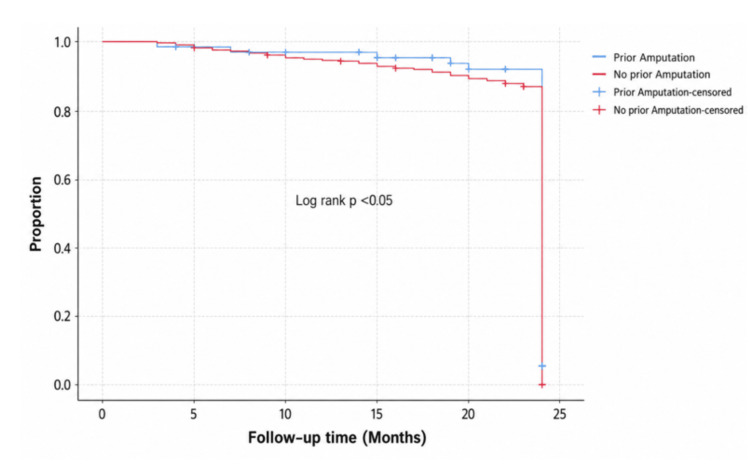
Kaplan-Meier estimates of freedom from major lower-extremity amputation over time (months), comparing patients with PAD with and without prior minor amputation. PAD, peripheral arterial disease

**Figure 2:**
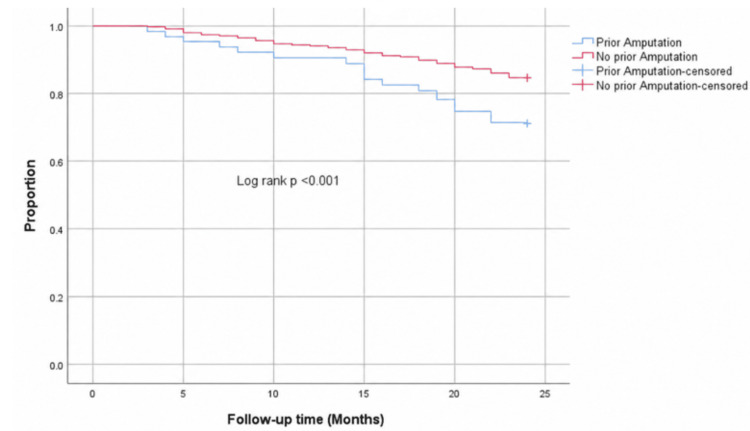
Kaplan-Meier estimates of overall survival (all-cause mortality) over the follow-up period (months), comparing patients with PAD with and without prior minor amputation. PAD, peripheral arterial disease

KM survival analysis demonstrated significant differences between groups (Figures 1-2). Figure 1 demonstrates the KM-estimated freedom from major lower-extremity amputation among patients with PAD, stratified by PMA status. The survival curves illustrate the cumulative probability of remaining free from major amputation over the 24-month follow-up period. At baseline, both groups exhibited comparable amputation-free survival, with survival probabilities close to 100%. Over time, a gradual decline in freedom from major amputation was observed in both cohorts; however, the PMA group consistently demonstrated a more favorable amputation-free profile compared with patients without PMA. Separation of the curves became more apparent during mid- to late follow-up, indicating differing long-term outcomes between the two populations. At 24 months, freedom from major amputation was higher in the PMA group than in the non-PMA group (93.0% vs. 88.9%), with a statistically significant difference in amputation-free survival (log-rank *P *< 0.05) (Figure 1). The survival curves separated progressively over time, with the non-PMA group showing a greater decline in amputation-free survival, especially during the later stages of follow-up. These findings suggest that PMA was not associated with worse limb-salvage outcomes after intervention and may reflect more intensive surveillance and follow-up in this patient population. Censoring events are represented by tick marks along the curves, reflecting patients who were lost to follow-up or remained event-free at the time of last assessment. Overall, the KM analysis demonstrated a significant divergence in major amputation-free survival between patients with PAD with and without PMA over the study period.

Figure 2 demonstrates the KM overall survival analysis among patients with PAD, stratified according to the presence or absence of PMA. The KM survival curves illustrate the cumulative probability of survival throughout the 24-month follow-up period. At the beginning of follow-up, survival probabilities were comparable between the two groups, remaining close to 100%. However, progressive divergence of the curves was observed over time, with patients in the PMA group demonstrating a steeper decline in survival compared with those without PMA. This separation became increasingly pronounced during later follow-up, indicating poorer long-term survival outcomes among patients with previous minor amputation. At 24 months, overall survival in the PMA group was 73.6%, significantly lower than the 89.7% observed in the non-PMA group. The difference in survival between the groups was statistically significant, as confirmed by the log-rank test (p<0.001), suggesting a strong association between PMA and reduced long-term survival in patients with PAD. Censoring events are indicated by tick marks along the survival curves and represent patients who remained alive at the time of last follow-up or were lost to follow-up during the study period. Overall, the KM analysis demonstrates significantly worse overall survival among patients with PAD with PMA compared with those without PMA over the 24-month follow-up duration.

## Discussion

This study demonstrates several key findings regarding outcomes in patients with PAD with and without PMA. First, patients with PMA exhibited distinct demographic and clinical profiles, characterized by higher rates of smoking, CKD, and revascularization procedures, yet paradoxically lower utilization of guideline-directed medical therapy [14,20]. Second, contrary to initial expectations, PMA was associated with significantly reduced risks of both major amputation and mortality. Third, medical therapies, including statins and anticoagulation, emerged as powerful protective factors for mortality, while revascularization showed an unexpected association with increased mortality risk [3,21].

The protective effect of PMA on subsequent major amputation (HR 0.05) is particularly striking and warrants careful interpretation. This finding aligns with observations from Barshes et al., who reported that minor amputation combined with palliative wound care did not result in worse survival outcomes compared to major amputation, while preserving ambulatory status and independent living in a greater proportion of patients [22]. Similarly, Birmpili et al. found that at one year after minor amputation in a large English cohort, the ipsilateral major amputation rate was only 10.7%, suggesting that many patients achieve limb salvage following minor amputation [14]. The protective effect may reflect successful source control of infection, removal of necrotic tissue, and optimization of wound healing potential in a more distal location, thereby preventing progression to more proximal amputation levels [5,16,22]. However, direct comparison with the present cohort should be approached with caution, as detailed measures of disease severity, such as chronic limb-threatening ischemia (CLTI) status, extent of tissue loss, wound, ischemia, and foot infection (WIFI) stage, and anatomic disease burden, were not consistently available in the current dataset. Therefore, differences in baseline disease severity may have influenced the observed associations.

Comparison with international studies reveals both similarities and important differences. European studies have consistently reported high mortality rates following major amputation [10,23,24,25]. Faglia et al. in Italy found that among diabetic patients with critical limb ischemia undergoing major amputation, 28.2% of revascularized patients and 81.2% of non-revascularized patients died, with revascularization significantly improving survival [24]. Klaphake et al. in the Netherlands reported overall mortality rates of 44%, 66%, and 85% at one, three, and five years, respectively, following major amputation in elderly patients with critical limb ischemia [25]. These mortality rates substantially exceed those in our cohort, potentially reflecting differences in patient age, disease severity, and healthcare system factors. Asian studies have also documented concerning outcomes. Morisaki et al. identified low serum albumin as a risk factor for 30-day mortality after major amputation in Japanese patients with PAD, highlighting the importance of nutritional status [11]. The Colombian study by Acero-Murillo et al. reported 14.7% overall mortality after major amputation, with significantly higher rates in acute ischemia (32.6%) compared to CLTI (11.2%) [26].

The observed association between revascularization and increased mortality (HR 2.02) requires cautious interpretation and should not be considered evidence of treatment-related harm. This finding likely reflects confounding by indication, wherein patients selected for revascularization had more severe disease, greater tissue loss, or a higher comorbidity burden compared to those managed conservatively [17,20,23]. Armstrong et al. demonstrated that conservative therapy without revascularization was associated with 2.08-times higher odds of major amputation or death compared to endovascular intervention, supporting the benefit of revascularization in appropriately selected patients [17]. The protective effects of statin therapy (HR 0.21) and anticoagulation (HR 0.39) on mortality align with extensive evidence supporting these therapies in PAD management and underscore the critical importance of optimal medical therapy [2,20].

The significantly lower utilization of antiplatelet therapy and statins in the PMA group (47.2% vs. 64.0% and 52.8% vs. 72.1%, respectively) represents a concerning gap in guideline-directed medical therapy [19]. Previous studies have linked high-intensity statin therapy to improved survival and reduced amputation risk in PAD populations [1,3,20]. As this was an observational study, these findings should be interpreted with caution, since PMA may reflect differences in disease progression, comorbidity burden, or survivor selection. The underutilization of these evidence-based therapies in patients with PMA - a population at particularly high risk - suggests an opportunity for quality improvement initiatives [27]. The higher rates of both open and endovascular revascularization in the PMA group (11.1% vs. 1.1% and 22.2% vs. 3.9%, respectively) likely reflect more advanced disease requiring intervention for limb salvage [17,23].

International trends support the increasing role of revascularization in PAD management. Nienaber et al. reported decreasing incidence of major amputations between 1990 and 2009 in a US population-based cohort, coinciding with increased utilization of revascularization procedures [28]. Malyar et al. in Germany found that revascularization rates before amputation increased from 46% in 2005 to 57% in 2009, though in-hospital mortality remained high at 19.8% for major amputations [23]. These findings emphasize that while revascularization is increasingly utilized, mortality outcomes remain suboptimal, highlighting the need for comprehensive risk factor modification and medical management in addition to procedural interventions [17,24,29,30].

The observed discrepancy between the unadjusted KM survival analysis and the adjusted Cox regression model likely reflects significant baseline differences between the study groups. In comparison to patients without PMA, the PMA group exhibited higher rates of smoking, CKD, and revascularization procedures, as well as lower use of antiplatelet and statin therapy. These variables are independently associated with adverse cardiovascular and limb-related outcomes and likely contributed to the poorer unadjusted survival observed in the PMA group. Adjustment for relevant covariates altered the direction of association, indicating that baseline confounding substantially influenced the unadjusted survival comparison.

Several limitations should be considered in interpreting these findings. The retrospective design introduces potential selection bias and restricts causal inference. The single-center setting may limit the generalizability of results to other healthcare environments. The relatively small sample size in the PMA group (*n* = 72) may have reduced statistical power to detect certain associations. Additionally, the limited number of outcome events may have increased the risk of model instability and overfitting in multivariable analyses. Employing a dichotomized age variable instead of modeling age as a continuous variable may have decreased statistical precision and resulted in residual confounding. The exceptionally low hazard ratio may be attributable to residual confounding, survivor bias, differences in baseline disease severity, treatment-selection effects, or model instability due to the limited number of outcome events. Consequently, this finding should be interpreted as hypothesis-generating rather than as evidence of a causal protective effect. Detailed data on anatomic disease severity, tissue loss classification, and specific revascularization techniques were unavailable for analysis. The use of multiple univariate comparisons may have increased the risk of type I error, raising the likelihood of identifying statistically significant associations by chance. Potential treatment imbalances between groups, including differences in medical therapy, revascularization strategies, wound care, or follow-up intensity, may have confounded the observed associations between PMA and clinical outcomes, further limiting causal inference. Some counterintuitive findings may reflect recruitment bias, as variations in patient selection, referral patterns, or baseline disease characteristics could have influenced the results and reduced generalizability.

## Conclusions

This study demonstrates that patients with PAD with PMA represent a distinct population with higher rates of smoking, CKD, and revascularization procedures, yet paradoxically lower utilization of guideline-directed medical therapies. In this retrospective cohort, PMA was associated with significantly reduced risks of subsequent major amputation and mortality, contrary to initial expectations. Statin therapy and anticoagulation were also associated with lower mortality. Although prior literature suggests that minor amputation and debridement may support limb-salvage strategies in selected patients with severe PAD, these findings should be interpreted as observational associations rather than evidence of a causal protective effect. Clinicians should prioritize aggressive medical management, particularly statin and antiplatelet therapy, in all patients with PAD regardless of amputation history. Future prospective studies are needed to validate the temporal and causal relationships between PMA and outcomes in PAD.
